# Effects of the Mitochondria-Targeted Antioxidant Mitoquinone in Murine Acute Pancreatitis

**DOI:** 10.1155/2015/901780

**Published:** 2015-03-23

**Authors:** Wei Huang, Nicole Cash, Li Wen, Peter Szatmary, Rajarshi Mukherjee, Jane Armstrong, Michael Chvanov, Alexei V. Tepikin, Michael P. Murphy, Robert Sutton, David N. Criddle

**Affiliations:** ^1^NIHR Liverpool Pancreas Biomedical Research Unit, Royal Liverpool University Hospital, UK; ^2^Department of Cellular and Molecular Physiology, University of Liverpool, Liverpool L69 3BX, UK; ^3^Department of Integrated Traditional Chinese and Western Medicine, Sichuan Provincial Pancreatitis Centre, West China Hospital, Sichuan University, China; ^4^Medical Research Council (MRC) Mitochondrial Biology Unit, Cambridge, UK

## Abstract

Although oxidative stress has been strongly implicated in the development of acute pancreatitis (AP), antioxidant therapy in patients has so far been discouraging. The aim of this study was to assess potential protective effects of a mitochondria-targeted antioxidant, MitoQ, in experimental AP using *in vitro* and *in vivo* approaches. MitoQ blocked H_2_O_2_-induced intracellular ROS responses in murine pancreatic acinar cells, an action not shared by the control analogue dTPP. MitoQ did not reduce mitochondrial depolarisation induced by either cholecystokinin (CCK) or bile acid TLCS, and at 10 *µ*M caused depolarisation *per se*. Both MitoQ and dTPP increased basal and CCK-induced cell death in a plate-reader assay. In a TLCS-induced AP model MitoQ treatment was not protective. In AP induced by caerulein hyperstimulation (CER-AP), MitoQ exerted mixed effects. Thus, partial amelioration of histopathology scores was observed, actions shared by dTPP, but without reduction of the biochemical markers pancreatic trypsin or serum amylase. Interestingly, lung myeloperoxidase and interleukin-6 were concurrently increased by MitoQ in CER-AP. MitoQ caused biphasic effects on ROS production in isolated polymorphonuclear leukocytes, inhibiting an acute increase but elevating later levels. Our results suggest that MitoQ would be inappropriate for AP therapy, consistent with prior antioxidant evaluations in this disease.

## 1. Introduction

Acute pancreatitis (AP) is a severe inflammatory condition of the exocrine pancreas caused primarily by gallstones and excess alcohol [1, 2] with an incidence of approximately 30 per 100,000 per year in the United Kingdom [3]. Although most patients have a mild and self-limiting clinical course [4] roughly 15–20% of cases involve potentially lethal complications such as persistent organ failure and infected pancreatic necrosis [5], resulting in a heavy socioeconomical burden [6]. Despite increased understanding of the pathophysiology of this disease in the last two decades, a specific therapy for AP is lacking [4].

The initial site of damage in AP is considered to be the pancreatic acinar cell, which exhibits pathological features including premature activation of digestive enzyme precursors, inhibition of apical secretion, disordered autophagy and lysosomal degradation, mitochondrial dysfunction, and release of inflammatory cytokines [7]. Evidence suggests a pivotal role for calcium, with diverse AP precipitants, such as bile acids, cholecystokinin hyperstimulation, and nonoxidative ethanol metabolites, inducing calcium overload, mitochondrial dysfunction, and loss of ATP that result in acinar cell necrosis [8], the extent of which determines outcome in a clinical setting. Localised and systemic inflammatory responses are features of AP progression, involving activation and infiltration of inflammatory cells into the pancreas provoking further injury in a vicious cycle [9].

A role for ROS has been proposed in the development of AP on the basis of an elevated oxidative status or reduced antioxidant capacity observed in the clinic and in experimental animal models [8, 10]. For example, increased superoxide and lipid peroxide levels and diminished antioxidant status were present in the blood of AP patients compared with healthy controls that correlated with disease severity [11]. Investigations in experimental AP, including* in vivo* caerulein hyperstimulation and bile acid models, have shown increases of ROS associated with the disease progression [12]. The involvement of ROS in AP is complex and poorly defined, with generation in the pancreatic acinar cell in response to many stimuli [13], including sustained calcium elevations induced by bile acid [14], and also by extrapancreatic inflammatory cells including neutrophils expressing NAD(P)H oxidase; knockout of this enzyme was associated with reduced severity of experimental AP [15]. Despite the strong evidence demonstrating oxidative stress in AP, there has been an apparent translational gap, with randomised clinical trials using antioxidant therapy so far discouraging [12].

A more recent strategy relating to antioxidant therapy has been to target compounds to the mitochondria in order to locally scavenge ROS and protect the organelle [16]. A mitochondrial-targeted antioxidant mitoquinone (MitoQ) has been designed to deliver a quinone antioxidant moiety to the mitochondria via a 10-carbon alkyl chain linked to a lipophilic cation triphenylphosphonium (TPP^+^) that utilises the mitochondrial membrane potential (ΔΨ*m*) for accumulation [17]. Studies have shown that MitoQ protects against many oxidative stress-induced conditions in cell lines and animal disease models, and this drug has undergone phase II clinical trials in hepatitis C patients [18]. However, potential protective effects of MitoQ in AP have not been tested so far. In this study we have investigated the effects of MitoQ on isolated pancreatic acinar cells, polymorphonuclear leukocytes (PMNs), and in two murine* in vivo* experimental AP models.

## 2. Materials and Methods

### 2.1. Animals and Reagents

Male CD1 mice (30–35 g) or C57BL/6J mice (20–25 g) were bought from Charles River UK Ltd. (Margate, UK). They were housed at 23 ± 2°C under a 12-hour light/dark cycle with ad libitum access to standard laboratory chow and water. For 12 hours before the start of the* in vivo* experiments, the animals were deprived of food but were allowed access to water. Studies were conducted in compliance with the appropriate UK Home Office personal and project licenses, and with the Institutional ethical review processes of the University of Liverpool. For reagents if not otherwise mentioned were all from Sigma (Gillingham, UK). MitoQ and its nonantioxidant control decyl-TPP (dTPP) were synthesised in the Department of Chemistry, University of Octago, New Zealand.

### 2.2. Isolation of Pancreatic Acinar Cells

Freshly isolated pancreatic acinar cells were obtained from the pancreas of adult CD1 mice using a standard collagenase (Worthington Biochemical Corporation, Lakewood, NJ, USA) digestion procedure established in previous work [19]. The extracellular solution contained (mM): 140 NaCl, 4.7 KCl, 1.13 MgCl_2_, 1 CaCl_2_, 10 D-glucose, and 10 HEPES. The final pH of the solution was adjusted to pH 7.35 using NaOH. All experiments on isolated pancreatic acinar cells were performed at room temperature (23–25°C) and the cells were used no more than 4 h after isolation if not otherwise stated.

### 2.3. Isolation of PMNs

Isolation of PMNs was achieved as previously described [20]. Murine long bones were isolated and flushed with sterile PBS, followed by filtered through a 70 mm sterile filter (Fisher Scientific, Loughborough, UK) and centrifuged at 600 g for 5 minutes at 4°C. The cell pellet was then suspended in 4 mL PBS and loaded onto a Percoll density gradient (62% and 81%), followed by centrifugation at 1500 g for 20 min at 4°C. Cells between Percoll layers were collected and suspended in 4 mL Red Cell Lysis Buffer for 5 min on a rocker, washed 2 : 1 with PBS, and centrifuged at 600 g for 5 min before suspending in RPMI1460. These isolated cells were further counted by a Countess Automated Cell Counter (Invitrogen, Carlsbad, CA, USA). All cells used in this study were morphologically >90% PMNs with >90% viability.

### 2.4. Measurement of ROS Production

Real-time ROS production and redox changes in pancreatic acinar cells were measured with the probe 5-chloromethyl-2,7-dichlorodihydrofluorescein diacetate acetyl (CM-H_2_DCFDA) using a Zeiss LSM510 confocal microscope (Carl Zeiss Jena GmbH, Jena, Germany) as previously described [14]. Freshly isolated murine pancreatic acinar cells were incubated with either 1 *μ*M MitoQ or dTPP and simultaneously loaded with 10 *μ*M CM-H_2_DCFDA for 30 minutes. The cells were then perfused with H_2_O_2_ to induce ROS. The fluorescence of CM-H_2_DCFDA was excited at 488 nm and the emission was collected at 505–550 nm.

For ROS measurement in PMNs, a peroxidase-enhanced luminol chemiluminescent assay was employed using a POLARstar Omega Plate Reader (BMG Labtech, Germany). Cells were plated at a density of 500,000 per well and pretreated with 1 *μ*M MitoQ or dTPP for 10 minutes, before adding 50 *μ*M luminol and 75 units/mL horseradish peroxidase. Activation of NAD(P)H oxidase was induced by 50 ng/mL phorbol myristate acetate (PMA) while inhibition was achieved by using 1 *μ*M diphenylene iodonium (DPI). The luminescence emission at 440 nm for the ROS dye was recorded for 40 min. The chemiluminescence intensity was normalised to negative controls for each mouse/run.

### 2.5. Measurement of ΔΨ*m* in Pancreatic Acinar Cells

In separate experiments, ΔΨ*m* of pancreatic acinar cells was determined by tetramethyl rhodamine methyl ester (TMRM; Molecular Probes, Eugene, OR, USA) assay as described previously [14]. Briefly, the cells were loaded with 40 nM TMRM for 30 minutes prior incubation with either 1 *μ*M or 10 *μ*M of MitoQ or dTPP. Cholecystokinin-8 (CCK-8, 10 nM) or bile acid taurolithocholic acid 3-sulphate (TLCS, 500 *μ*M) was used to induce ΔΨ*m* depolarisation. At the end of the perfusion, the protonophore carbonyl cyanide 3-chlorophenylhydrazone (CCCP, 10 *μ*M) was added to induce complete depolarisation of ΔΨ*m*. The fluorescence of TMRM was excited at 543 nm and the emission was collected at 560–650 nm.

### 2.6. *In Vitro* Cell Death Assay for Pancreatic Acinar Cells

Pancreatic acinar cell death was detected as the intensity of fluorescent dye propidium iodide (PI) taken up by the nuclei of necrotic cells [14]. For cell death induced by CCK-8, a time-course fluorescent plate reader method was used. Briefly, cells were isolated from one murine pancreas, centrifuged, and resuspended into 1 mL solution. Cells were carefully pipetted into individual wells to ensure homogeneity. Cells were treated with CCK-8 (10 nM) alone, or in the presence of 1 *μ*M of either MitoQ or dTPP. For normal control groups, cells treated with equal volume of extracellular solution. After 5 min, PI (50 *μ*M) was then added to all wells mixed by automated agitation. The microplate was then placed in the POLARstar Omega Plate Reader (preheated to 37°C), and fluorescence determined by excitation 543 nm and emission 620 nm with bottom reading. The assay was set to run with a cycle time of 600 seconds. All fluorescence measurements are expressed as changes from basal fluorescence (*F*/*F*_0_ ratio), where* F*_0_ represents the initial fluorescence recorded at the start of the experiment, and* F* the fluorescence recorded at specific time points.

### 2.7. Experimental AP Models

Seven intraperitoneal injections of a supramaximal dose (50 *μ*g/kg) of caerulein, a CCK-8 analogue, were given on an hourly basis to induce hyperstimulation acute pancreatitis (CER-AP) [21]. Control mice received equal volumes of PBS injection. In the MitoQ treatment groups, MitoQ at 10 mg/kg (dose 1) or 25 mg/kg (dose 2) was given at the first and third injections of caerulein. Similarly, dTPP at 9.6 mg/kg (dose 1) or 24 mg/kg (dose 2) was given for the dTPP treatment group. MitoQ and dTPP were at the same molar concentration at doses 1 and 2. Mice were sacrificed at 12 h after the first caerulein injection to collect samples.

Bile acid-induced AP was achieved by retrograde infusion of TLCS into the pancreatic duct (TLCS-AP) [22]. After induction of anesthesia, TLCS applied using a mini infusion pump (Harvard Apparatus, Kent, UK) at a speed of 5 *μ*L/min for 10 minutes. Successful infusion of TLCS into pancreas was demonstrated by a diffuse light blue colour (methylene blue) appearing in the pancreatic head. Control mice received sham surgery without TLCS infusion. In the treatment groups, MitoQ (10 mg/kg) or dTPP (9.6 mg/kg) was given at 1 h and 3 h after TLCS infusion. Mice were sacrificed at 24 h after the TLCS infusion or sham surgery. In both animal models, analgesia was achieved by administration of 0.1 mg/kg buprenorphine hydrochloride.

### 2.8. Severity Markers for AP

After sacrifice of the mice, blood was allowed for natural clotting for 30 minutes and followed by centrifugation at 1,500 g × 10 minutes to collect serum. Pancreas and lung samples were also harvested and snap frozen for future use. A proportion of pancreas was also fixed by 10% formalin overnight before being subjected to H&E staining (5 *μ*m thick per slide).

Serum amylase was tested in Clinical Biochemistry Department in Royal Liverpool University Hospital using a kinetic method. Serum IL-6 was measured by ELISA according to manufacturer's instruction (R&D, Abingdon, UK). Pancreatic trypsin activity was determined by established protocol using trypsin peptide substrate Boc-Gln-Ala-Arg-MCA (Peptide, Osaka, Japan) on a fluorescent plate reader (BMG Labtech, UK). Pancreatic and lung myeloperoxidase activity, pancreatic trypsin activity, and pancreatic histopathology were analysed by methods previously reported [23]. All histopathological scoring was undertaken in a double-blinded manner by independent assessors.

### 2.9. Statistical Analysis

Results were presented as mean ± SEM obtained from three or more independent experiments. In all the figures, vertical bars denote SEM values. A Student's* t*-test was used for statistical evaluation of data with a normal distribution, while an ANOVA test was carried out for data with a skewed distribution.* P* values of <0.05 were considered to indicate significant differences.

## 3. Results 

### 3.1. MitoQ Scavenged ROS Production in Isolated Pancreatic Acinar Cells

**Figure 1:**
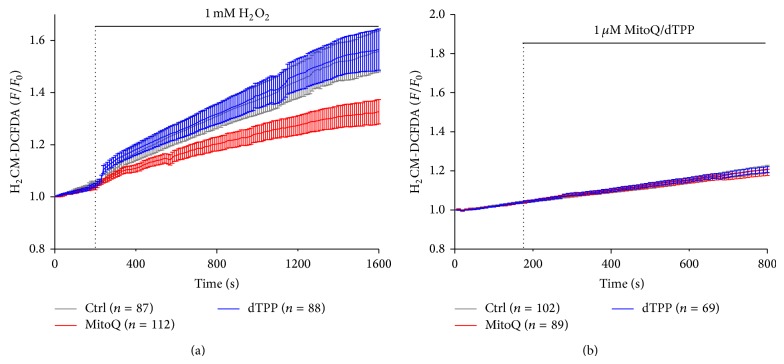
Effects of MitoQ on ROS responses in isolated pancreatic acinar cells. (a) 1 mM H_2_O_2_ induced a steady rise of intracellular ROS production, indicated by an increase in DFDCA fluorescence, which was significantly reduced by 1 *μ*M MitoQ but not dTPP. (b) Neither 1 *μ*M MitoQ nor dTPP caused ROS production* per se*. (*n* ≥ 3 mice per group). DCFDA fluorescent traces are shown as normalised (*F*/*F*_0_) mean ± SEM.

Application of 1 mM H_2_O_2_ caused a steady rise of ROS in the cells (control group) as reflected by increased intensity of CM-H_2_DCFDA fluorescence; Cells pretreated with 1 *μ*M MitoQ showed a significantly reduced ROS production compared with cells in the control group, whereas pretreatment of 1 *μ*M dTPP, the lipophilic cation of MitoQ without antioxidant activity was without effect (Figure 1(a)). Neither MitoQ nor dTPP induced ROS production* per se* (Figure 1(b)).

### 3.2. MitoQ Did Not Protect against Mitochondrial Depolarisation Caused by AP Precipitants in Isolated Pancreatic Acinar Cells

**Figure 2:**
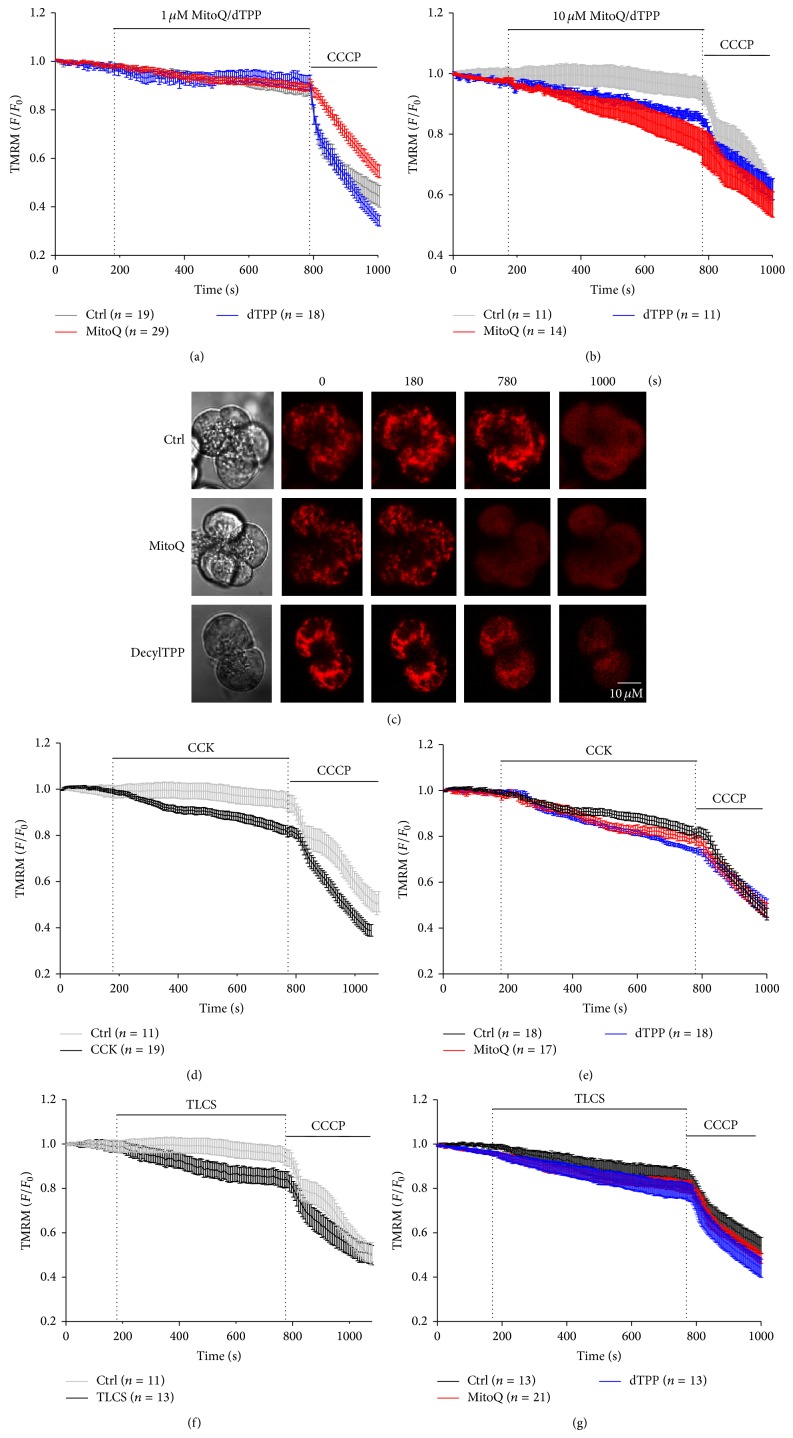
Effects of MitoQ on mitochondrial membrane potential (ΔΨ*m*) in isolated pancreatic acinar cells. (a) Neither 1 *μ*M MitoQ nor dTPP had a significant effect on ΔΨ*m per se*. In contrast the protonophore CCCP induced complete depolarisation. Mean data (b) and representative images (c) showing that 10 *μ*M MitoQ and dTPP partially depolarised mitochondria. Application (d) CCK (10 nM) or (f) TLCS (500 *μ*M) caused a fall of ΔΨ*m* compared to time-matched controls. However, neither 1 *μ*M MitoQ nor dTPP pretreatment protected against ΔΨ*m* depolarisation induced by (e) CCK (10 nM) or (g) TLCS (500 *μ*M) (*n* ≥ 3 mice per group). TMRM fluorescent traces are shown as normalised (*F*/*F*_0_) mean ± SEM.

Neither pretreatment with 1 *μ*M MitoQ nor dTPP caused depolarisation of ΔΨ*m* in isolated pancreatic acinar cells, in contrast to the protonophore CCCP applied at the end of the experiment to elicit complete depolarisation. (Figure 2(a)). However, at 10 *μ*M both MitoQ and dTPP induced a steady decrease of ΔΨ*m per se* that was more profound for MitoQ (Figures 2(b) and 2(c)). CCK (10 nM) induced depolarisation of ΔΨ*m* compared with control cells treated with HEPES alone (Figure 2(d)), an effect that was not significantly affected by pretreatment with either 1 *μ*M MitoQ or dTPP (Figure 2(e)). Similarly, perfusion of TLCS (500 *μ*M) depolarised ΔΨ*m* (Figure 2(f)) that was unaffected by 1 *μ*M MitoQ or dTPP (Figure 2(g)).

### 3.3. MitoQ Caused Pancreatic Acinar Cell Death and Aggravated CCK-Induced Necrosis

**Figure 3:**
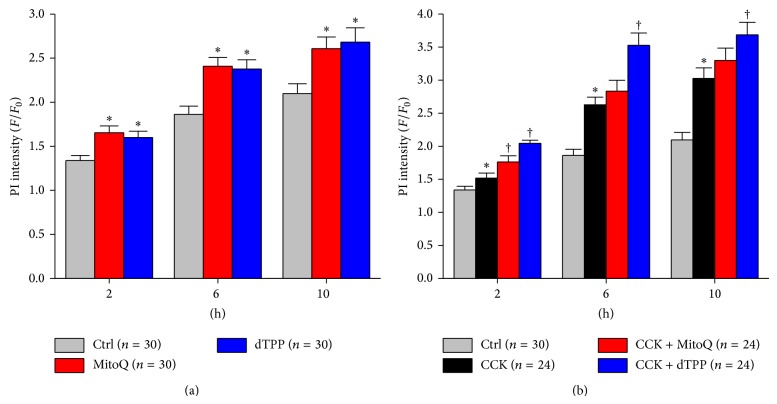
Effects of MitoQ on cell death in isolated pancreatic acinar cells. (a) Both 1 *μ*M MitoQ and dTPP caused significant increases in necrosis, demonstrated by an increase in PI fluorescence compared with cells in the control group. (b) Both 1 *μ*M MitoQ and dTPP worsened CCK-induced cell death. ^*^ *P* < 0.05 compared with control group, ^†^compared with CCK group. (*n* ≥ 3 mice per group).

Both 1 *μ*M MitoQ and dTPP caused an increased PI uptake indicative of necrosis; significant differences were observed at 2, 6, and 10 h between cells pretreated with either MitoQ or dTPP and cells in the control group treated with HEPES alone (Figure 3(a)). Addition of 10 nM CCK induced a time-dependent increase of necrosis. However, 1 *μ*M MitoQ did not exert any protection against CCK-induced cell death. Rather both MitoQ and dTPP significantly worsened necrosis compared with CCK alone at 2 h, whereas dTPP, but not MitoQ, aggravated CCK-induced cell death at later time-points (Figure 3(b)).

### 3.4. MitoQ Ameliorated Overall Pancreatic Histopathology in CER-AP but Aggravated Systemic Injury

**Figure 4:**
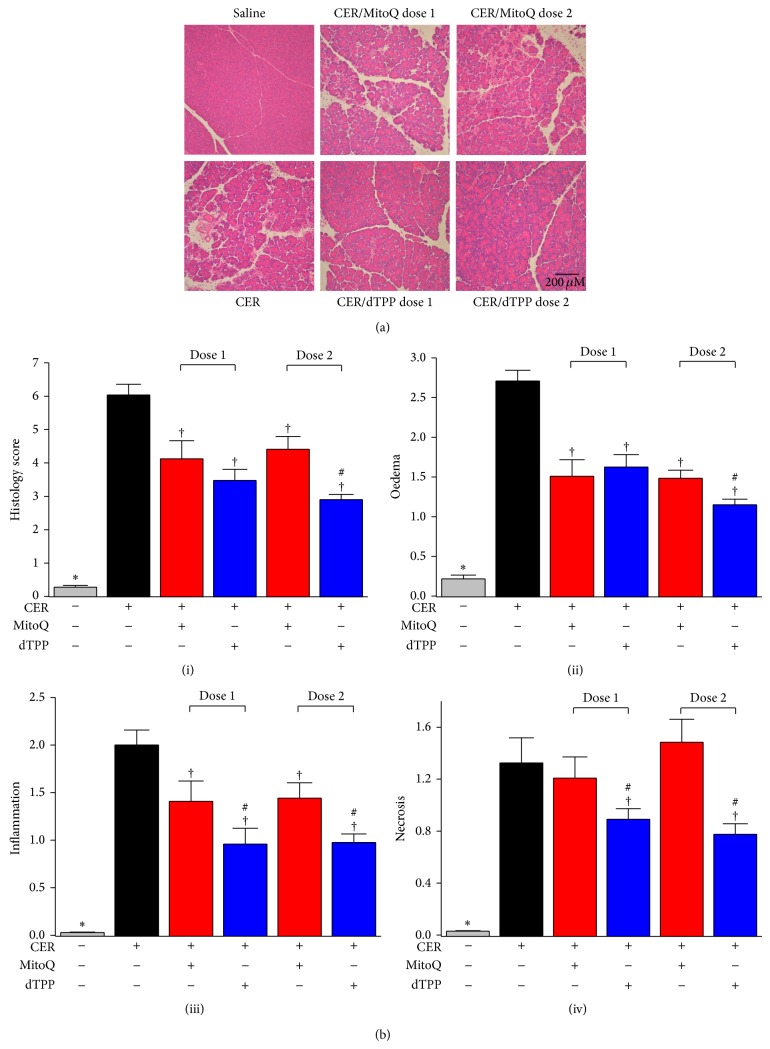
Effects of MitoQ on histopathological changes of CER-AP. (a) Representative H&E images for all experimental groups (magnification 200x). (b) Histopathology scores: (i) overall, (ii) oedema, (iii) inflammation and (iv) necrosis. ^*^ *P* < 0.05 when compared with all other groups, ^†^compared with CER-AP alone group, ^#^compared with MitoQ group at the same dose. Values are the mean ± SEM (*n* = 6 mice per group).

Figure 4(a) shows representative histopathology slides for control and different treatment groups, with the overall histopathology score and breakdown scores for individual components summarised in Figure 4(b). Intraperitoneal saline injections did not cause any significant histopathological changes of the pancreas, whereas hyperstimulation with caerulein induced typical features of AP; marked oedema, vacuolisation, neutrophil infiltration in the ductal margins, and parenchyma of the pancreas, with focal acinar cell necrosis evident 12 h after the first caerulein injection. The CER-AP was also characterised by significantly increased serum amylase, pancreatic trypsin and MPO activity, and lung MPO activity compared to saline controls.

**Figure 5:**
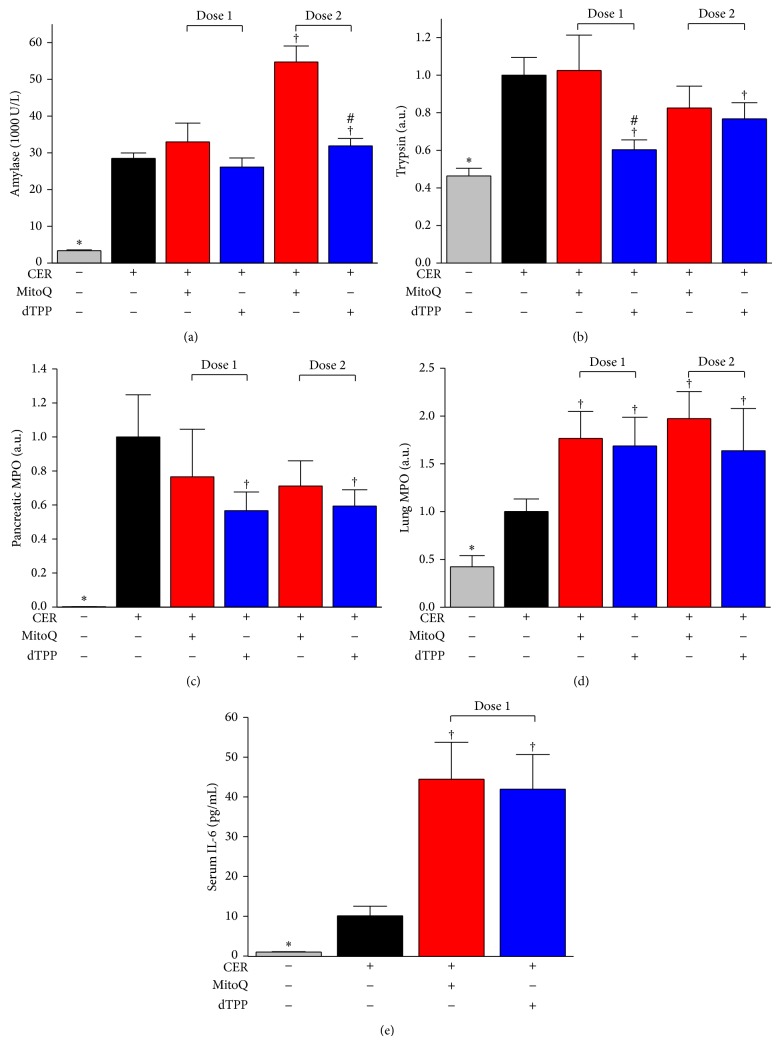
Effects of MitoQ on biochemical markers of CER-AP. (a) Serum amylase. (b) Pancreatic trypsin activity (normalised to CER-AP group). (c) Pancreatic and (d) lung MPO activity (both normalised to CER-AP group). (e) Serum IL-6 levels. ^*^ *P* < 0.05 when compared with all other groups, ^†^compared with CER-AP group, ^#^compared with MitoQ group at the same dose. Values are the mean ± SEM (*n* = 6 mice per group).

MitoQ treatment at both doses tested significantly reduced pancreatic oedema and neutrophil infiltration. However, pancreatic necrosis was not prevented, with a trend toward greater necrosis at the higher dose although this did not attain significance. MitoQ dose-dependently increased serum amylase with an approximate doubling at the higher dose (Figure 5(a)). Pancreatic trypsin activity and MPO activity were not significantly affected by MitoQ at either dose (Figures 5(b) and 5(c)). In addition, MitoQ treatment nearly doubled lung MPO activity induced by caerulein (Figure 5(d)) with a significant increase of serum IL-6 levels also evident at dose 1 (Figure 5(e)).

The nonantioxidant analogue dTPP significantly reduced oedema, neutrophil infiltration, and necrosis at both doses, resulting in an overall reduction of the histopathological score. Serum amylase was not significantly affected (Figure 5(a)), although dTPP reduced pancreatic trypsin and MPO activity (Figures 5(b) and 5(c)). Similar to the results obtained with MitoQ, dTPP also significantly increased caerulein-induced lung MPO activity and serum IL-6 levels (Figures 5(d) and 5(e)).

### 3.5. MitoQ Did Not Protect against TLCS-AP

**Figure 6:**
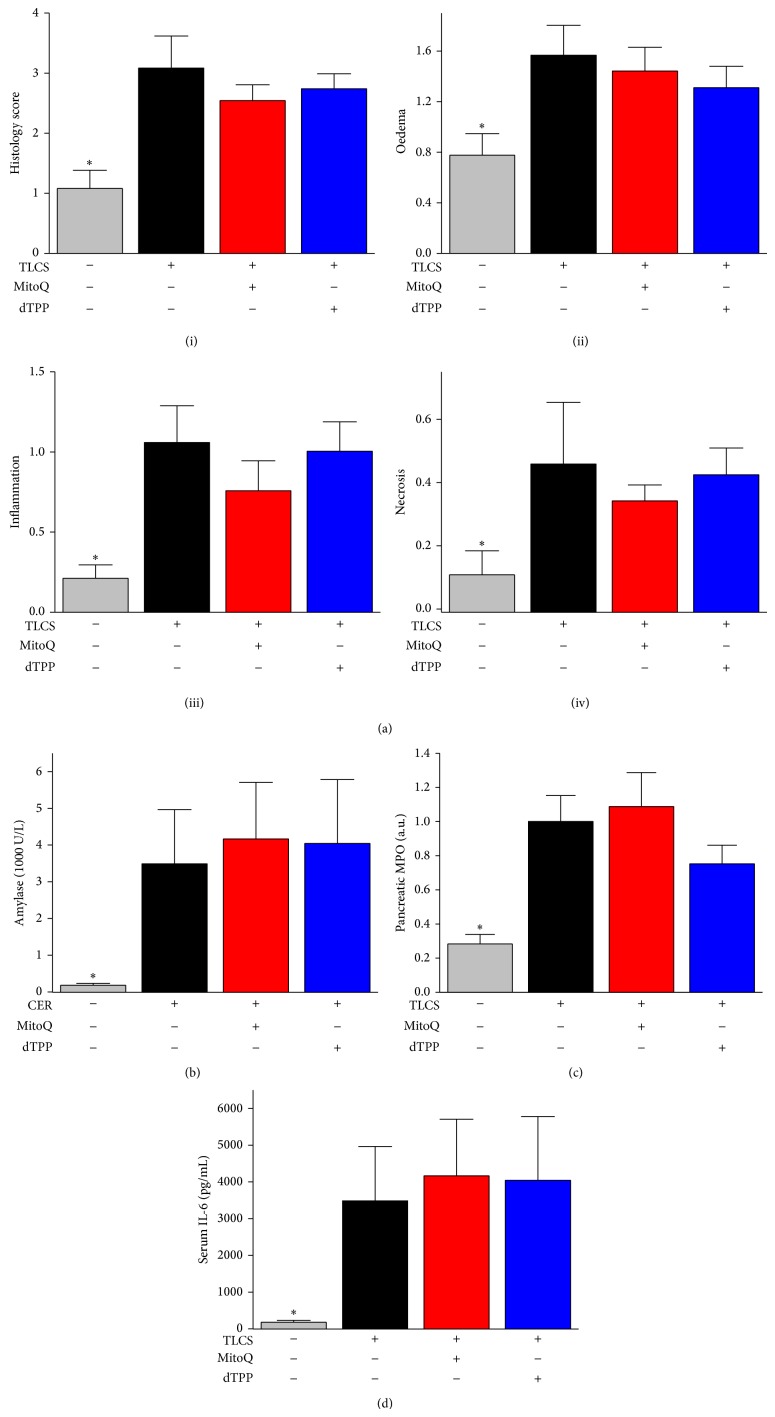
Effects of MitoQ on TLCS-AP. (a) Histopathology scores: (i) overall, (ii) oedema, (iii) inflammation, and (iv) necrosis. (b) Serum amylase. (c) Pancreatic MPO activity (normalized to CER-AP group). (d) Serum IL-6 levels. ^*^ *P* < 0.05 when compared with all other groups. Values are the mean ± SEM (*n* = 6–8 mice per group).

Sham operation only induced mild oedema of the pancreatic acinar cells without discernible signs of inflammation and necrosis. Infusion of 3 mM TLCS into the pancreas via the pancreatic duct resulted in marked histopathological changes of the head of the pancreas at 24 h, characterised by significantly increased oedema, inflammation, necrosis and thus, overall histopathology score (Figure 6(a)(i–iv)). However, the body and tail of the pancreas were much less affected (data not shown). The TLCS-AP was associated with increased serum amylase, pancreatic MPO activity, and serum IL-6 levels compared to the sham group (Figures 6(b)–6(d)).

Neither MitoQ nor dTPP at the lower dose induced histopathological changes of the pancreas, with oedema, inflammation, necrosis, and overall histopathological score unaltered (Figure 6(a)(i–iv)). Similarly, there were no significant changes of serum amylase and pancreatic MPO activity when TLCS-AP mice were treated with either MitoQ or dTPP (Figures 6(b) and 6(c)). Serum IL-6 levels were marginally increased by MitoQ or dTPP treatment but this did not attain statistical significance (Figure 6(d)). Application of MitoQ or dTPP at both doses alone to mice in the absence of caerulein injections or TLCS infusion showed that both MitoQ and dTPP significantly increased lung MPO activity* per se* (data not shown).

### 3.6. Biphasic Effects of MitoQ on PMA-Induced ROS Production in PMNs

**Figure 7:**
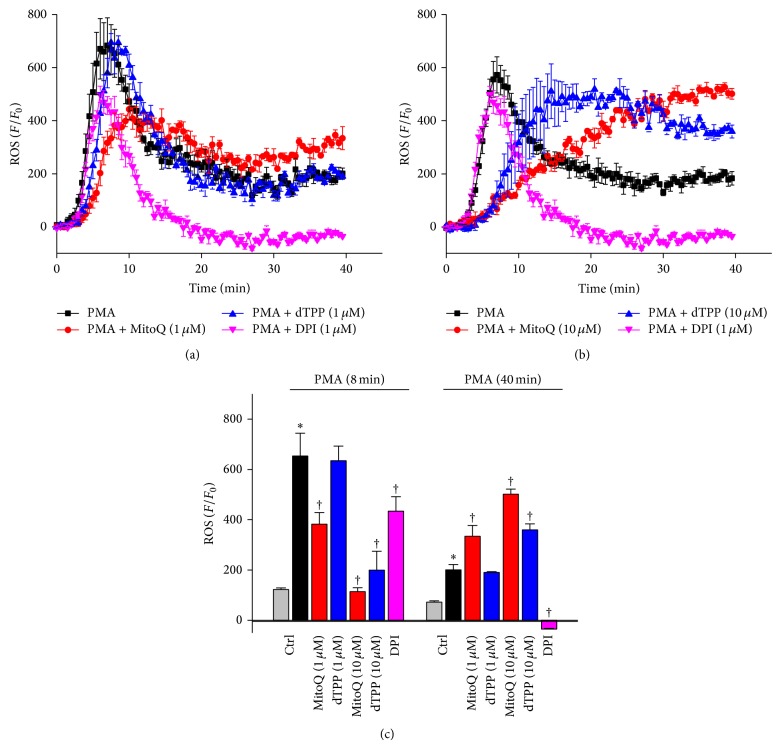
Effects of MitoQ on PMA-induced ROS production in isolated PMNs. (a) Effects of 1 *μ*M MitoQ or dTPP and 1 *μ*M DPI on ROS production induced by 50 ng/mL PMA. (b) Effects of 10 *μ*M MitoQ or dTPP and 1 *μ*M DPI on ROS production induced by 50 ng/mL PMA. (c) Summary bar charts for PMA-induced ROS production at 8 mins (peak) and 40 mins for all experimental groups. ^*^ *P* < 0.05 when compared with control groups and ^†^compared with PMA alone group. Values are the mean ± SEM (*n* ≥ 3 mice per group).

Figure 7(a) illustrates the effect of 1 *μ*M MitoQ or dTPP on PMA-induced ROS production in isolated PMNs. The NAD(P)H oxidase stimulator PMA (50 ng/mL) induced a dramatic increase of ROS in the extracellular solution around PMNs within the first few minutes that peaked at 8 mins and declined to a plateau after approximately 20 mins. Application of the NAD(P)H oxidase inhibitor DPI reduced the peak phase and completely inhibited the ROS plateau (Figure 7(c)). MitoQ treatment caused a biphasic effect on ROS production in PMNs. Thus, a concentration-dependent inhibition of the initial ROS peak induced by PMA was observed, with the peak time delayed to 10 minutes (Figures 7(a)–7(c)). Application of 1 *μ*M dTPP had no significant effect on the peak of PMA-induced ROS production in PMNs compared to cells treated by PMA alone. Interestingly, MitoQ caused a concentration-dependent potentiation of PMA-induced ROS production at 40 mins (Figure 7(c)), an action shared by dTPP only at the higher concentration.

## 4. Discussion 

Our study has evaluated, for the first time, the effects of a mitochondria-targeted antioxidant MitoQ on* in vitro *and* in vivo* models of experimental AP. Previous preclinical evaluations of antioxidants in AP models have produced mixed results, with unsuccessful translation to the clinic thus far [12]. A confounding factor that might have positively influenced prior outcomes of antioxidant evaluations in experimental models is that such agents have mostly been administered as a pretreatment; that is, before induction of the disease, that does not adequately reflect the clinical situation. Therefore in the present study MitoQ was given as a treatment after the induction of AP in two experimental models. CER-AP is one of the most commonly used animal models of AP but has few clinical parallels [7], whereas TLCS-AP mimics gallstone aetiology with necrotising pancreatitis in the pancreatic head associated with moderate to severe systemic manifestations, including greatly elevated serum IL-6.

MitoQ has been shown to exert protective effects in diverse disease models that are associated with oxidative stress, including colitis [24], encephalomyelitis [25], diabetes [26], cardiac ischaemia-reperfusion injury [27], and sepsis [28]. However, the actions of MitoQ in the two murine experimental models of AP were complex, with TLCS-AP unaffected by treatment with the mitochondrial antioxidant. Although no studies have directly measured oxidative stress in mouse TLCS-AP, elevated markers have been demonstrated in the pancreas and erythrocytes following pancreatic ductal taurocholate administration in rats [29–31]. The study of Rau and colleagues [30] indicated that whilst ROS might be mediators of tissue damage, their extracellular generation alone did not induce typical biochemical and morphological changes indicative of AP; a lack of a protective effect of MitoQ in the current TLCS-AP model would support this view. In contrast, the general antioxidant N-acetylcysteine (NAC), which prevented oxidant-induced ROS increases in pancreatic acinar cells [32], reduced tissue necrosis, leukocyte infiltration, oedema, and haemorrhage in taurocholate-induced AP in rats, although this was given as pretreatment rather than postinsult [33]. However, an investigation in murine CER-AP specifically compared the effects of NAC administration before and after the first caerulein injection; only the prophylactic treatment was successful in limiting the severity of experimental AP whereas antioxidant therapy postinsult was ineffective [34].

In the present study some protective actions of MitoQ were evident in the CER-AP model, although effects were variable and shared by the nonantioxidant control dTPP. Thus, MitoQ partially protected against the severity of CER-AP as assessed by pancreatic histopathology, but without a significant reduction of pancreatic necrosis, increased in isolated pancreatic acinar cells. No reduction of serum amylase or pancreatic trypsin was evident, whilst MitoQ concurrently elevated systemic injury markers such as lung MPO activity and serum IL-6. In addition, perhaps surprisingly, dTPP significantly improved overall and individual pancreatic histopathology scores, decreased pancreatic trypsin, and reduced pancreatic MPO activity. Currently the explanation for any beneficial effect of dTPP is unclear, but some deleterious effects of MitoQ may have resulted from antioxidant activity. Previously we have shown that ROS inhibition in isolated pancreatic acinar cells exposed to TLCS results in more necrosis and less apoptosis, indicating a protective role for ROS in these cells [14]. At higher doses both MitoQ and dTPP caused mitochondrial depolarisation* per se* and increased pancreatic acinar cell death, possibly indicative of nonspecific toxic effects that might relate to uncoupling actions due to accumulation of fatty acyl chains in the mitochondria. Fatty acids are known to uncouple oxidative phosphorylation and previously we have shown that application of long-chain fatty acids to isolated pancreatic acinar cells caused mitochondrial depolarisation, loss of NAD(P)H and ATP, leading to necrosis [35, 36]. In the present study both MitoQ and dTPP augmented basal and CCK-induced necrosis in a cell death assay.

Interestingly, MitoQ exerted biphasic effects on ROS production in PMNs, generated by activation of NAD(P)H oxidase. Thus, it initially inhibited the PMA-induced acute ROS peak at 8 mins but later potentiated the plateau at 40 mins. This initial inhibition was not shared by dTPP, suggesting that it was attributable to the ROS scavenging properties of MitoQ. In contrast, the NAD(P)H oxidase inhibitor DPI, which similarly reduced acute ROS levels, also completely abolished the late phase. The PMNs, which account for about 60% of leukocytes, are essential for innate immunity and one of earliest inflammatory cells to arrive at the infection/injury site [37]. In the experimental AP models, histology indicated that PMNs accumulated moderately within the pancreas 2 hours after induction and were abundant at 6 hours. In clinical acute pancreatitis, enrolment and infiltration of PMNs in the pancreas and distant organs are a principal feature of the disease [38]. PMNs may further aggravate tissue injury by releasing ROS that are generated by NAD(P)H oxidase [15] or by degranulation and release of their nuclear contents to form extracellular traps [39]. Serum IL-6, mainly secreted by myeloid cells including PMNs, is a cytokine known to connect pancreatic injury to distal organ damage [40] and also serves as severity marker for human AP [41]. Thus, an overall increase in ROS production in the PMNs induced by MitoQ and dTPP may have facilitated lung MPO, generating hypochlorous acid and reactive oxidants, further enhancing its activity. Indeed, both MitoQ and dTPP at the doses used for the* in vivo* experiments significantly increased lung MPO activity* per se* (data not shown).

## 5. Conclusion

In conclusion, the findings of this study further emphasize the unsuitability of antioxidant therapy in the treatment of AP, previously highlighted by a randomised, double-blind, and placebo-controlled clinical trial [42]. There was no protection of experimental TLCS-AP by MitoQ and mixed effects observed in the milder CER-AP model, including elevations of inflammation markers. These results are in accordance with previous studies showing that suppression of ROS enhances pancreatic acinar cell necrosis by inhibiting a protective apoptotic mechanism [14], an action that would promote local pancreatic damage in AP.
